# Gold-Based Metal Drugs as Inhibitors of Coronavirus Proteins: The Inhibition of SARS-CoV-2 Main Protease by Auranofin and Its Analogs

**DOI:** 10.3390/biom12111675

**Published:** 2022-11-11

**Authors:** Lara Massai, Deborah Grifagni, Alessia De Santis, Andrea Geri, Francesca Cantini, Vito Calderone, Lucia Banci, Luigi Messori

**Affiliations:** 1 Department of Chemistry “Ugo Schiff”, University of Florence, Via Della Lastruccia 3, 50019 Florence, Italy; 2 Magnetic Resonance Center (CERM), University of Florence, Via L. Sacconi 6, Sesto Fiorentino, 50019 Florence, Italy; 3 Consorzio Interuniversitario Risonanze Magnetiche Metallo Proteine (CIRMMP), University of Florence, Via L. Sacconi 6, 50019 Florence, Italy

**Keywords:** COVID-19, nsp5, M^pro^, SARS-CoV-2, Auranofin, gold compounds, ESI mass spectrometry

## Abstract

Gold compounds have a long tradition in medicine and offer many opportunities for new therapeutic applications. Herein, we evaluated the lead compound Auranofin and five related gold(I) complexes as possible inhibitors of SARS-CoV-2 Main Protease (SARS-CoV-2 M^pro^), a validated drug target for the COVID-19 disease. The investigational panel of gold compounds included Auranofin; three halido analogues, i.e., Au(PEt_3_)Cl, Au(PEt_3_)Br, and Au(PEt_3_)I; and two gold carbene complexes, i.e., Au(NHC)Cl and [Au(NHC)_2_]PF_6_. Notably, all these gold compounds, with the only exception of [Au(NHC)_2_]PF_6_, turned out to be potent inhibitors of the catalytic activity of SARS-CoV-2 M^pro^: the measured K_i_ values were in the range 2.1–0.4 μM. The reactions of the various gold compounds with SARS-CoV-2 M^pro^ were subsequently investigated through electrospray ionization (ESI) mass spectrometry (MS) upon a careful optimization of the experimental conditions; the ESI MS spectra provided clear evidence for the formation of tight metallodrug-protein adducts and for the coordination of well defined gold-containing fragments to the SARS-CoV-2 M^pro^, again with the only exception of [Au(NHC)_2_]PF_6_, The metal-protein stoichiometry was unambiguously determined for the resulting species. The crystal structures of the metallodrug- M^pro^ adducts were solved in the case of Au(PEt_3_)Br and Au(NHC)Cl. These crystal structures show that gold coordination occurs at the level of catalytic Cys 145 in the case of Au(NHC)Cl and at the level of both Cys 145 and Cys 156 for Au(PEt_3_)Br. Tight coordination of gold atoms to functionally relevant cysteine residues is believed to represent the true molecular basis of strong enzyme inhibition.

## 1. Introduction

The outbreak and the rapid spread of COVID-19 disease are posing dramatic problems to health systems worldwide [1]. The identification and rapid implementation of effective antiviral drugs against SARS-CoV-2 are urgently needed to fight this severe disease. To this end, expanding the chemical space of the tested compounds by including a large variety of metal compounds is highly desirable. It has to be stressed that the search for efficient inhibitors of SARS-CoV-2-related proteases has almost entirely focused on organic molecules [2,3,4]; much less attention has been deserved so far to inorganic moieties [5,6] that merit, in our opinion, a greater consideration.

Indeed, the known inorganic drugs contain a wide array of metals imparting peculiar chemical properties, that arise from the electronic structure of the metal center, its coordination sphere, the nature of the ligands, the redox properties, etc. It is evident that these chemical features cannot be completely reproduced by simple organic compounds. Accordingly, the unique chemical and biological properties of the various metal centers- in many cases non-physiological metals- should be taken into due account within new drug discovery programs. This approach might hopefully lead to successful pharmacological and therapeutic outcomes.

The main protease of the SARS-CoV-2 virus is nsp5, also known as 3CL^pro^ or M^pro^. This is a homodimeric protein with each monomer having a molecular mass of 33,796 Da. Each monomer contains three domains. More in detail, domain I (residues 8–101) and domain II (102–184) fold in an antiparallel β-barrel -where the active site with the Cys145-His41 catalytic dyad is located [7] whereas domain III (residues 201–306) is involved in the process of dimerization that is critical for the function of the enzyme [8]. The two subunits of M^pro^ bind to each other via the N-terminus (residues 1–7) where the Ser1 of each monomer completes and stabilizes the S1 substrate binding pocket of the adjacent monomer [9]. The M^pro^ function is indispensable for viral replication as it cleaves the polyproteins pp1a and pp1ab at eleven conserved sites, to allow the correct folding and function of essential viral proteins [10,11]. For this reason, M^pro^ is a widely accepted pharmacological target [12]. It is also important to consider that M^pro^ is an enzyme with a high structural conservation of the main core among the various coronaviruses [13,14]; therefore, the discovery of high-affinity inhibitors for this protease may be an excellent starting point for selecting new antiviral compounds that are active against a broad spectrum of coronaviruses [15]. The recently solved 3D structures of SARS-CoV-2 M^pro^ in complex with zinc ion [16,17,18] showed that the metal ion is bound to the catalytically relevant Cys145 and His 41 residues. Furthermore, a few recent works demonstrated that other metal ions or metal-based compounds, such as rhenium, gold and copper compounds, can be efficient inhibitors of viral proteases [19,20,21]. For instance, Auranofin is a representative gold drug that was claimed to exhibit remarkable antiviral properties; notably, the efficacy of Auranofin and some related gold compounds against Papain-like protease was nicely documented by Ingo Ott et al. [20]. In addition, the crystal structure of the adduct formed between SARS-CoV-2 M^pro^ and Auranofin was recently solved showing that gold is, at least partially, able to target the catalytic cysteine residue [22]. Another recent work [23] has analyzed an even larger set of gold compounds; this study proved once more the ability of such compounds to inhibit the catalytic activities of SARS-CoV-2 M^pro^ and papain-like protease (PL^pro^).

Here we show, through in-vitro enzymatic activity assays, the ability of three gold(I) Auranofin analogs and two gold carbene complexes to inhibit the SARS-CoV-2 M^pro^ activity, and compare the results with those obtained with Auranofin. All of these gold complexes as already shown interesting cytotoxicity properties and antimicrobial activities [24,25,26], then, using the approach of the repurposing strategy we selected this panel of compounds to identify new possible drugs for the treatment of the SARS-CoV-2 disease. Moreover, we show, through mass spectrometry analysis, that these gold(I) compounds bind SARS-CoV-2 M^pro^ tightly forming well-defined metal-protein complexes. In addition, the crystallographic 3D structures for two of these metal-protein complexes have been solved providing atomic-level details about the binding mode of the gold(I) centers to the protein and disclosing the molecular basis for the observed enzyme inhibition. Summarizing, all the above experiments reveal a significant affinity of gold(I) compounds for active site thiols in SARS-CoV-2 M^pro^. The resulting inhibition constants are estimated in the order of the low micromolar range, which is indeed a promising starting point for the design of new and potent gold-based inhibitors of SARS-CoV-2.

## 2. Materials and Methods

### 2.1. Sources of Compounds and Chemicals

The M^pro^ was produced through heterologous expression in *E. coli* as previously described in [16]. The gold compounds were prepared and characterized according to the previously reported procedures [27,28].

The fluorescence-quenched peptide substrate used in the enzymatic assay has been purchased from GenScript Biotech, Leiden, Netherlands. If not otherwise specified, reagents and chemicals used have been purchased from Merck KGaA, Darmstadt, Germany.

### 2.2. Enzymatic Activity Assay

An initial stock of M^pro^ 10 μM in 20 mM Tris-HCl, 150 mM NaCl 1 mM EDTA, 5 mM DTT pH 7.8 was diluted in the measurement buffer (Tris-HCl 20 mM 20% glycerol pH 7.2) up to 0.2 μM. This solution was used to prepare different samples containing increasing concentrations of metal-based drugs as follows: Auranofin (0, 0.5, 1, 2, 4, 6, 8, 10, 12 and 15 µM,), Au(NHC)Cl (0, 0.3, 0.6, 1, 2, 3, 6, 8, 10, 15 and 20 µM), Au(PEt_3_)Br (0, 0.1, 0.2, 0.3, 0.4, 0.6, 0.8, 1, 1.5, 2, 3 and 6 µM), Au(PEt_3_)I (0, 0.5, 1, 1.5, 2, 3, 4, 5, 6, 8, 10 and 15 µM) and Au(PEt_3_)Cl (0, 0.1, 0.25, 0.5, 0.75, 1, 1.25, 1.5, 2, 2.5, and 3 µM). The protein-molecule solutions were incubated at r.t. for 2 h, thereafter 4 μM of a fluorescence-quenched peptide substrate (Mca–AVLQ↓SGFR-K(Dnp)K) was added for each measurement. Fluorescence curves were acquired for 1 min at an excitation and emission wavelength of 320 and 405 nm, respectively. All experiments were performed in duplicate at least two times. The fluorescence curve slopes were calculated by linear fits at 18 s of the fluorescence signal increase as a function of time. For each molecule, all measurements were analyzed in a single fitting, which allowed the calculation of K_i_ values using the Hill equation where Vmax and Vmin were fixed at 100 and 0, respectively, while the Hill coefficient, n, was not fixed. Originpro 2018 was used to analyzed all the spectra.

## 3. Electrospray Ionization Mass Spectrometry Experimental Conditions

### 3.1. Sample Preparation

Stock solutions of SARS-CoV-2 M^pro^ were prepared by dissolving the protein in ammonium acetate solution 2  ×  10^−3^ M (pH 6.8), mixed with aliquots of DTT in cysteine-to-reducing agent ratio 1:5. Stock solutions 10^−2^ M of the gold(I) compounds were prepared by dissolving the compounds in DMSO. For the experiments with SARS-CoV-2 M^pro^, aliquots of the respective stock solutions were mixed with aliquots of gold compounds at a protein-to-metal ratio of 1:3 and diluted with ammonium acetate solution 2 × 10^−3^ M (pH 6.8) to 10^−4^ M final protein concentration. In the case of Au(PEt_3_)Br, the protein-to-metal ratio was 1:1. The mixtures were incubated at 37 °C up to 24 h. After the incubation time, all solutions were sampled and diluted to a final protein concentration of 10^−6^ M using ammonium acetate solution 2 × 10^−3^ M, pH 6.8.

### 3.2. Instrumental Parameters

The ESI mass study was performed using a TripleTOF 5600  +  high-resolution mass spectrometer (AB Sciex, Framingham, MA, USA), equipped with a DuoSpray^®^ interface operating with an ESI probe. The general ESI source parameters optimized for protein analysis were as follows: SARS-CoV-2 M^pro^: positive polarity, ion spray voltage floating 5500 V, temperature 0 °C, ion source Gas 1 (GS1) 25 L/min; ion source Gas 2 (GS2) 0; curtain gas (CUR) 45 L/min, collision energy (CE) 10 V; declustering potential (DP) 300 V, acquisition range 2400–5000 *m/z*. For acquisition, Analyst TF software 1.7.1 (AB Sciex, Framingham, MA, USA) was used, and deconvoluted spectra were obtained by using the Bio Tool Kit micro-application v.2.2 embedded in PeakViewTM software v.2.2 (AB Sciex, Framingham, MA, USA).

### 3.3. Crystallization, Data Collection and Structure Solution

Crystals of apo SARS-CoV-2 M^pro^ were obtained in sitting drop by adding an aliquot of 2 µL of protein solution (20 mM Tris-HCl, 150 mM NaCl, 1 mM EDTA, 1 mM DTT, pH 7.8) to 2 µL of reservoir buffer (20 mM ammonium acetate, 20% PEG 3350 pH 7) and stored at 20 °C. The protein concentration in the sample was 5 mg/mL. The native crystals of apo SARS-CoV-2 M^pro^ were afterwards soaked in a 10% DMSO solution containing Au(PEt_3_)Br Au(NHC)Cl respectively having a 2–3-fold ligand concentration with respect to the protein for about 20 h. Both adduct datasets were collected in-house, using a BRUKER D8 Venture diffractometer equipped with a PHOTON III detector, at 100 K; the crystals used for data collection were cryo-cooled using 25% ethylene glycol in the mother liquor. The crystals diffracted up to 2.2–2.3 Å resolution but structures have been refined at 2.4 Å: they belong to space group C2 with one molecule in the asymmetric unit, a solvent content of about 50%, and a mosaicity of 0.6°. The data were processed using the program XDS [29] reduced and scaled using XSCALE [29] and amplitudes were calculated using XDSCONV [29]. Both structures have been solved using the molecular replacement technique; in both cases, the model used was the 7NXH structure. The successful orientation hand translation of the molecule within the crystallographic unit cell was determined with MOLREP [30]. The refinement and water molecule fitting has been carried out using PHENIX [31], applying default TLS restraints. In between the refinement cycles, the model was subjected to manual rebuilding using COOT [32]. The quality of the refined structures was assessed using the program MOLPROBITY [33]. Data processing and refinement statistics for both adducts are shown in Table S1. Coordinates and structure factors have been deposited at the PDB under the accession code 8B0T for the Au(PEt_3_)Br and 8B0S for Au(NHC)Cl.

## 4. Results and Discussion

### 4.1. Chemical Features of the Study Compounds

The screening of drugs already approved for a different disease (the so-called “drug repurposing”) may result in an excellent drug discovery strategy, particularly in case a new therapeutical approach needs to be rapidly developed against an emerging disease. This strategy may allow to skip safety assessment, preclinical testing, and formulation development, i.e., the long and laborious phases that are typically required for the approval of a newly synthesized drug, thus drastically reducing the times for clinical implementation [34]. In this regard, Auranofin could be a very good candidate for drug repurposing. In fact, this drug was approved for the treatment of rheumatoid arthritis in 1988 and its safety profile is well-known and acceptable; Auranofin is currently under investigation for cancer therapy as a repurposed drug [35] and has also been proposed to fight a number of bacterial, parasitic and viral infections [36]. The potential of Auranofin as a drug for the treatment of SARS-CoV-2 recently emerged as a hot topic; a few studies demonstrated its ability to inhibit viral replication by contrasting specific enzymatic activities of the virus [5,6,37]. Auranofin could perform its antiviral activity in different ways: one of these is blocking the entry of the virus into the host cell; another is to inhibit the activity of selected viral enzymes that are crucial for virus replication and a last one is to counter host inflammatory response [38].

On the contrary, the other gold compounds presented herein, have not been approved yet for clinical use, but have been extensively tested for cellular cytotoxicity against a variety of cancer cells. Their IC_50_ values [39,40] typically fall in the low-micromolar range thus revealing the opportunity to be further investigated in cells and in vivo as prospective anticancer agents. In addition, some of these compounds may be exploited for different therapeutic uses, e.g., as antiviral or antimicrobial agents, if given at sub-cytotoxic concentrations.

**Figure 1:**
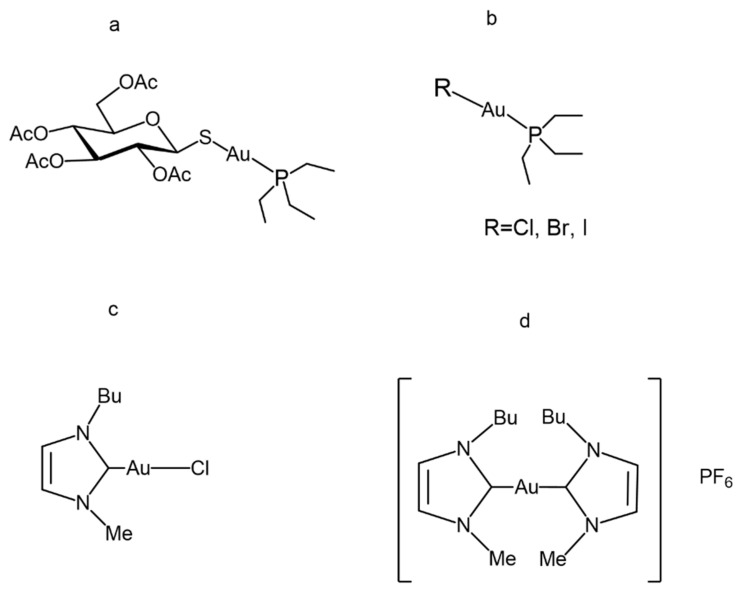
Gold(I) compounds chemical structure: (**a**) Auranofin, (**b**) Auranofin halido analogues, (**c**) Au(NHC)Cl and (**d**) [Au(NHC)_2_]PF_6._

The panel of gold compounds that we have used for the present investigation is shown in Figure 1. The panel contains six distinct gold(I) compounds, namely Auranofin, three halido analogs, i.e., Au(PEt_3_)Cl, Au(PEt_3_)Br and Au(PEt_3_)I, and two gold carbene compounds, i.e., Au(NHC)Cl and [Au(NHC)_2_]PF_6_. All these compounds contain a linearly coordinated gold(I) center and were previously described and characterized [27,28]. In four cases, i.e., Au(PEt_3_)Cl, Au(PEt_3_)Br, Au(PEt_3_)I, and Au(NHC)Cl, the halido ligand behaves as the leaving group; the sugar ligand is the leaving group in the case of Auranofin. Activation of the gold(I) center in [Au(NHC)_2_]PF_6_ is far more difficult as it requires the removal of a strong carbene ligand.

### 4.2. Enzyme Inhibition by the Study Compounds

**Figure 2:**
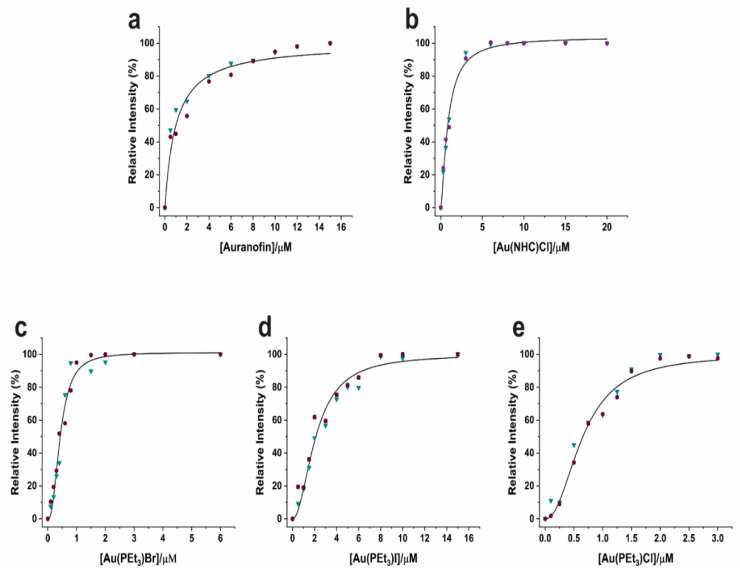
Inhibition M^pro^ profiles by (**a**) Auranofin (**b**) Au(NHC)Cl (**c**) Au(PEt_3_)Br (**d**) Au(PEt_3_)I (**e**) Au(PEt_3_)Cl. Two independent measurements are shown as red dots and blue triangles, respectively. K_i_ values were determined by nonlinear regression.

The ability of the various gold compounds to inhibit the catalytic activity of SARS-CoV-2 M^pro^ was comparatively measured using a previously established procedure [16]. A dose-dependent M^pro^ inhibition assay was carried out by treating 0.2 µM solutions of M^pro^ with increasing concentrations of the various panel compounds (ranging from 0 to 20 µM; Figure S1). All the experiments were performed in duplicate and the initial rates of the reactions were measured through the linear fittings of the fluorescence curves. The resulting inhibition profiles are shown in Figure 2, whereas the values of the inhibition constants, K_i_, are reported in Table 1. Notably, as all tested gold compounds, with the only exception of [Au(NHC)_2_]PF_6_, give a K_i_ value in the low µM range, they may be reputed as promising inhibitors of this enzyme.

Among the panel compounds, Au(PEt_3_)Br exhibited the maximum inhibitory potency for M^pro^; the inhibitor potencies of Au(PEt_3_)Cl, Au(PEt_3_)I and Au(NHC)Cl were roughly similar, and close to the value of Auranofin. [Au(NHC)_2_]PF_6_ turned out to be a very weak inhibitor of M^pro^ activity causing no more than 10% loss of activity at the very high concentration of 60 μM (Figure S2).

### 4.3. Metallodrug-Enzyme Interactions Probed by ESI MS

**Figure 3:**
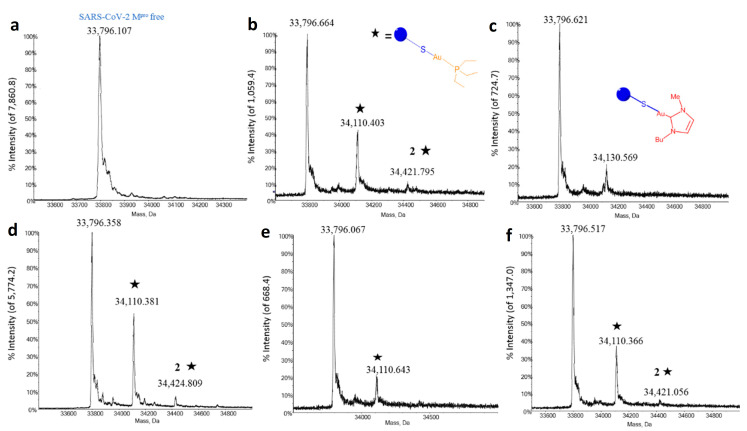
Deconvoluted ESI-QTOF mass spectra of M^pro^ incubated with different gold compounds for 4 h, (**a**) M^pro^ free (**b**) Auranofin (**c**) Au(NHC)Cl (**d**) Au(PEt_3_)Br (**e**) Au(PEt_3_)I (**f**) Au(PEt_3_)Cl. The star indicates the compound moiety that is bound to the protein in the case of Auranofin and its derivates.

To better explain the above inhibition profiles further studies have been carried out to characterize in detail the metallodrug-M^pro^ interactions that form the molecular basis of enzyme inhibition. Thus, the interactions between all panel compounds and the enzyme were investigated through ESI MS experiments. In principle, ESI MS is an excellent tool to characterize protein metalation by metal-based drugs. Indeed, a number of successful studies were reported in the recent literature [41,42,43,44,45,46] However, in the case of M^pro^, we encountered a lot of experimental difficulties in setting properly the ESI MS experiments to characterize the SARS-CoV-2 M^pro^/ gold metallodrug adducts. A lot of efforts were done to determine the optimal conditions for recording the ESI MS spectra; the best conditions that were eventually identified and then applied to the various samples are described in the methods section. The resulting ESI MS spectra offer clear evidence that SARS-CoV-2 M^pro^ may bind metallic fragments originating from the various gold drugs upon activation (Figure 3) as described below.

Notably, the deconvoluted mass spectrum of the free protein shows a well-resolved signal at 33796 Da that nicely matches the molecular mass of the native protein. Upon mixing, Auranofin and its halido analogues manifest a good reactivity with SARS-CoV/2-M^pro^ already after 4 h of incubation, giving rise to two different signals, respectively assigned to the mono- and the (generally less abundant) bis-adduct; in both cases, the metal-bound fragment nicely corresponds to the [Au(PEt_3_)]^+^ moiety (MW 315). Similarly, the interaction between the monocarbene gold complex Au(NHC)Cl and the protein results in the formation of a mono adduct bearing the 1-butyl-3-methylimidazole-2-ylidene-gold(I) moiety (MW 336) (Figure 3b). In all cases, the signal of unreacted SARS-CoV/2-M^pro^ is still present, indicating that the metalation of the protein is not complete under the applied experimental conditions. According to previous literature and previous experience of our laboratory on similar systems [43], it may be proposed that the resulting gold fragments will bind preferentially to solvent-exposed cysteines of the M^pro^. Instead, in accord with the less reactive character of the bis-gold(I) carbene complex, no metalation of the protein was observed upon reaction with [Au(NHC)_2_]PF_6_ even after 24 h of incubation, and just the free protein signal was invariably detected (Figure S2). This confirms that [Au(NHC)_2_]PF_6_ is poorly reactive with this protein in line with its scarce enzyme-inhibiting activity.

### 4.4. Crystallographic Results

**Figure 4:**
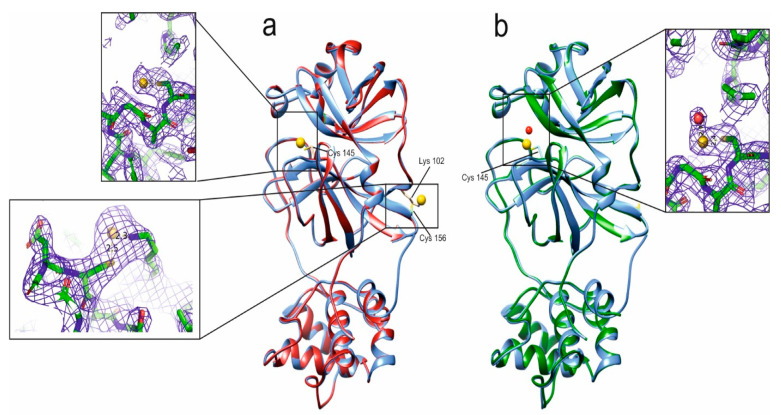
Overlays of (**a**) apo M^pro^ (cyan), (PDBID 7NXH), with M^pro^-Au(PEt_3_)Br adduct (red) and (**b**) apo M^pro^ (cyan) with M^pro^- Au(NHC)Cl adduct (dark green). Gold ion is shown as yellow sphere and the water molecule as red sphere. Cys 145, Cys 156 and Lys 102 are shown as sticks. Insets show the 2Fo-Fc electron density contoured at 1 s level of Au(PEt_3_)Br Cys145, Au(PEt_3_)Br Cys156 and Au(NHC)Cl Cys145 coordination environments.

Remarkably, for two of the above-mentioned gold drugs, i.e., Au(PEt_3_)Br and Au(NHC)Cl, we succeeded in obtaining good quality crystals of their adducts with M^pro^ and in solving the respective 3D crystal structures. The polypeptide structure of both adducts is totally superimposable with the others already deposited on the PDB except for the last five C-terminal residues that have not been modeled due to their very weak electron density. The structure of both SARS-CoV-2 M^pro^ adducts clearly resembles that of the apo protein (PDBID 7NXH) with a negligible RMSD computed on backbone atoms. Only local loop regions showed backbone RMSD values higher than average (Figure 4).

To the best of our knowledge, the structures of only two M^pro^/gold adducts are available on the PDB (7DAT and 7DAU [22]). In the 7DAT structure, M^pro^ has been soaked with Auranofin; the ionic gold targets the catalytic Cys145 with an occupancy of about 0.33 and Cys156 with an occupancy of 0.16 (Figure S3). In the 7DAU structure, ionic gold is bound to both Cys145 and Cys156 with occupancy values of 0.2 and 0.1, respectively. The distance between gold and sulfur in 7DAU is around 2.3 Å. The behavior of each of the two gold adducts analyzed in this work is slightly different, but they share a quite low gold occupancy, with the organic moiety originally attached to gold being no more visible in all the structures. In the case of Au(PEt_3_)Br, both Cys145 and Cys156 are involved in the binding of gold (7DAT and 7DAU structures, Figure 4A and Figure S3). The electron density of gold bound to Cys156 is quite well defined with an occupancy of about 0.4 and a B-factor in the range of the solvent atoms (Inset of Figure 4A). On the contrary, the gold ion bound to Cys145 has a weaker electron density due to a significantly lower occupancy that is as low as about 0.15. Concerning the coordination geometry, Cys145 binds gold in a monodentate fashion whereas, in the case of Cys156, gold shows a peculiar binding mode to the cysteine sulfur and to the terminal nitrogen of the Lys102 sidechain (insets of Figure 4A).

In the case of Au(NHC)Cl, only Cys145 is involved in the binding to gold but still with an occupancy of about 0.15, and a gold-sulfur distance of about 2.4 Å, which is in line with the expected values. At variance with Auranofin, a labile water molecule coordinates the gold ion in this complex (Figure 4B).

## 5. Conclusions

In conclusion, we have shown that Auranofin and its halido analogs behave as potent and effective inhibitors of the M^pro^ of SARS-CoV-2. Furthermore, while Au(NHC)Cl results to have inhibitory capabilities comparable to the Auranofin analogues, the gold dicarbene complex [Au(NHC)_2_]PF_6_ was found to inhibit the M^pro^ rather weakly in accordance with the mass data offering no evidence of adduct formation with the protein. The peculiar behavior of [Au(NHC)_2_]PF_6_ may be ascribed to the great strength of the two Au-C bonds and to the intrinsic difficulties in breaking them. Notably, the measured inhibition constants for Auranofin, for its three halido derivatives, and for AuNHC all fall in the low micromolar range. As SARS-CoV-2 M^pro^ is a validated druggable target of the coronavirus, these strong inhibitory properties suggest that the gold compounds studied in this work may be prospective anti-COVID-19 agents. ESI MS measurements point out that relatively stable metallodrug-protein adducts are indeed formed when mixing the protein with the various metal compounds through the coordination of gold-containing fragments to accessible protein residues in accordance with previous studies on similar systems; clear evidence is offered for the formation of adducts bearing one or two protein-bound gold fragments. In two cases, i.e., the protein adducts with Au(PEt_3_)Br and Au(NHC)Cl, high-resolution crystal structures have been obtained allowing unambiguous localization of the gold binding sites. Crystal structures show that Cys 156 and the catalytic Cys 145 are the main sites of protein metalation. Overall, the here reported results coherently support the view that the potent and promising inhibition of the catalytic activity of M^pro^ produced by these gold compounds is the likely consequence of the tight coordinative binding of a gold-containing fragment to the catalytic Cys 145. As a final consideration, it has to be pointed out that there are currently no in vivo experimental data concerning the ability of the gold compounds described in this paper to inhibit the SARS-CoV-2 virus. Within this frame, a possible future development of our study would be to explore the ability of such gold compounds to contrast SARS-CoV-2 infection in in vivo models and to compare their mechanism of action and their pharmacological efficacy with that of the reference gold drug Auranofin.

## Figures and Tables

**Table 1:** Inhibition constants, K_i_ values, measured for the interaction between gold compounds and M^pro.^

Compound	K_i_
Auranofin	0.99 ± 0.107 µM
Au(NHC)Cl	0.87 ± 0.04 µM
Au(PEt_3_)Br	0.44 ± 0.022 µM
Au(PEt_3_)I	2.12 ± 0.096 µM
Au(PEt_3_)Cl	0.66 ± 0.026 µM

## Data Availability

Coordinates and structure factors of the two crystallographic structures described in the paper are publicly available at the Protein Data Bank under the accession codes 8B0T for the Au(PEt_3_)Br and 8B0S for Au(NHC)Cl.

## Supplementary Materials

The following supporting information can be downloaded at: https://www.mdpi.com/article/10.3390/biom12111675/s1, Figure S1: Fluorescence curves of M^pro^ titration with (a) Auranofin (b) Au(NHC)Cl (c) Au(PEt_3_)Br (d) Au(PEt_3_)I (e) Au(PEt_3_)Cl; Figure S2: Activity assay raw data showing the absence of inhibition by [Au(NHC)_2_]PF_6_ and ESI-MS spectrum of a 1:1 M^pro^: [Au(NHC)_2_]PF_6_ solution showing no adduct formation; Figure S3: Superposition between Au(PEt_3_)Br red, 7DAT (Auranofin) magenta, Au(NHC)Cl green. Gold ions are represented as spheres and colored in yellow. Cys 145 and Cys156 are shown as sticks; Table S1: Data collection and refinement parameters.

## Abbreviations

DTT (dithiothreitol), EDTA (ethylenediaminetetraacetic acid), DMSO (Dimethylsulfoxide), PEG (polyethyleneglycole), ESI MS (Electrospray ionization mass spectrometry), PDB (protein data bank), RMSD (root mean square deviation)
