# Supplementary material for: Gold-Based Metal Drugs as Inhibitors of Coronavirus Proteins: The Inhibition of SARS-CoV-2 Main Protease by Auranofin and Its Analogs
Source: Biomolecules. 2022 Nov 11;12(11):1675. doi: 10.3390/biom12111675 (PMC9687241; doi:10.3390/biom12111675)
Supplement: Supplementary file 1 [file biomolecules-12-01675-s001.zip › biomolecules-1978604-supplementary.pdf]

Gold metallodrugs to target coronavirus proteins: the inhibition of SARS CoV-2 main protease by Auranofin and its analogs.

Lara Massai[*1], Deborah Grifagni[*1,2], Alessia De Santis[1,2], Andrea Geri[1], Francesca Cantini[1,2,3,±], Vito Calderone[1,2,3±], Lucia Banci[1,2,3], Luigi Messori[1±]

1 Department of Chemistry "Ugo Schiff", University of Florence, Via della Lastruccia 3 50019 Florence, Italy

2 Magnetic Resonance Center (CERM), University of Florence, Via L. Sacconi 6 50019 Sesto Fiorentino, Italy

3 Consorzio Interuniversitario Risonanze Magnetiche Metallo Proteine (CIRMMP), University of Florence, Via L. Sacconi 6 50019 Florence, Italy;

Correspondence to:

[±] Francesca Cantini: cantini@cerm.unifi.it

ORCID: 0000-0003-0526-6732

[±]Vito Calderone: calderone@cerm.unifi.it

ORCID: 0000-0002-7963-6241

[±]Luigi Messori: luigi.messori@unifi.it

ORCID: 0000-0002-9490-8014

This PDF file includes:

Figure S1 to S3

Table S1

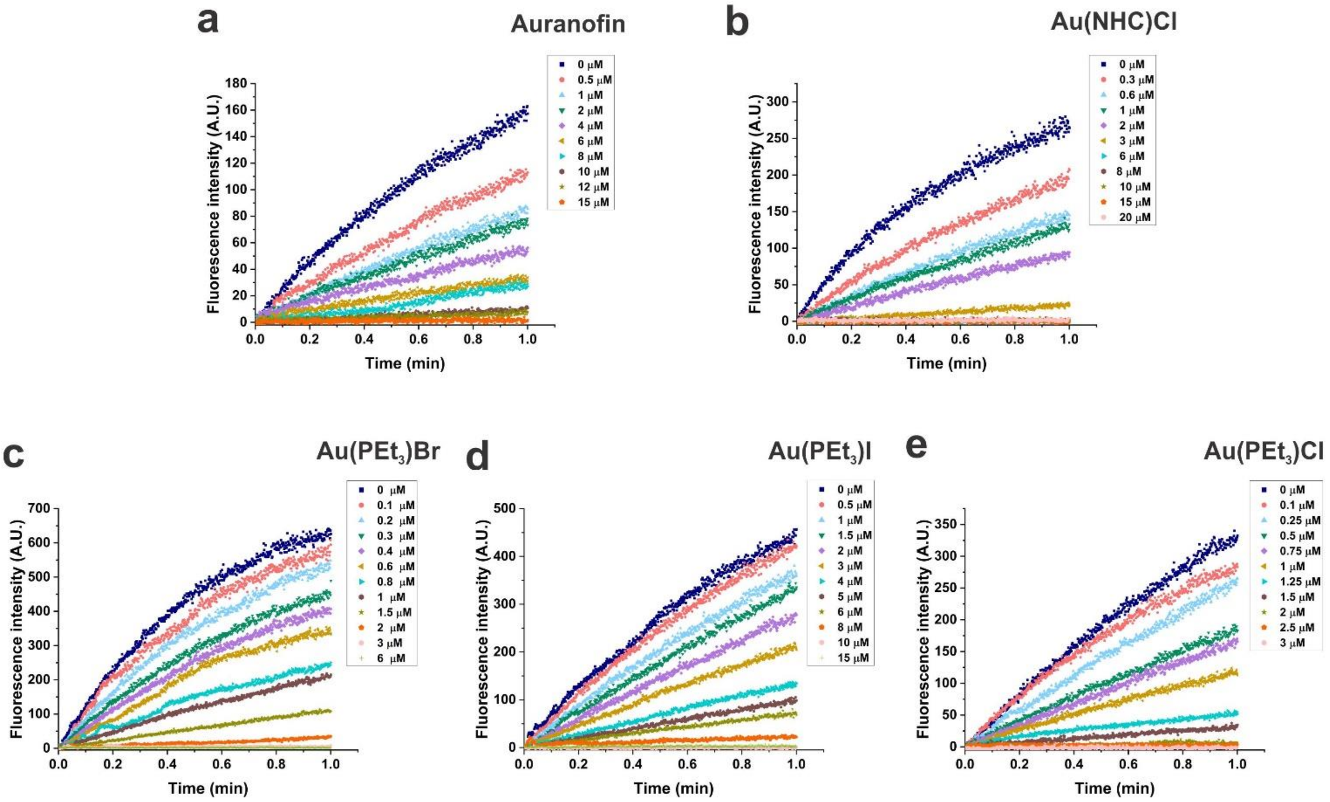

Figure S1. Fluorescence curves of M^pro titration with a) Auranofin b) Au(NHC)Cl c) Au(Pet$_3$)Br d) Au(PEt$_3$)I e) Au(Pet$_3$)Cl.

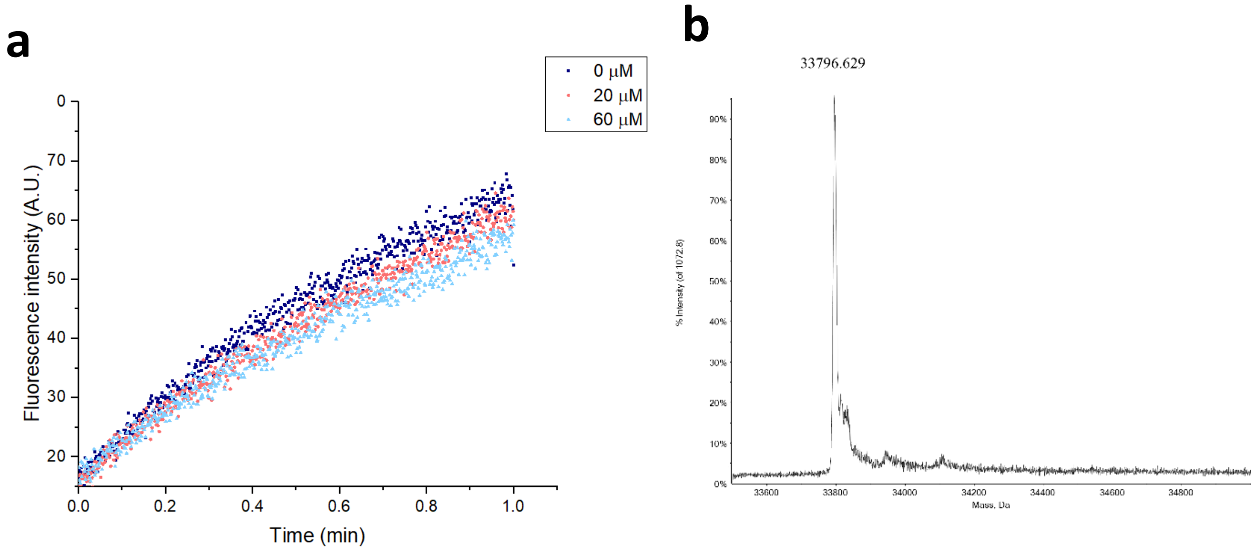

Figure S2. Activity assay raw data showing the absence of inhibition by [Au(NHC)$_2$]PF$_6$ and ESI-MS spectrum of a 1:1 M^pro: [Au(NHC)$_2$]PF$_6$ solution showing no adduct formation.

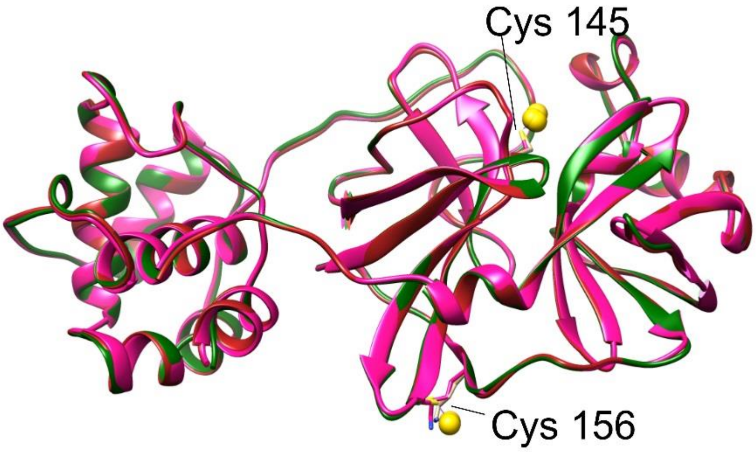

Figure S3. Superposition between Au(PEt_3)Br red, 7DAT (Auranofin) magenta, Au(NHC)Cl green. Gold ions are represented as spheres and colored in yellow. Cys 145 and Cys156 are shown as sticks.

Table S1. Data collection and refinement parameters.

	$Au(PEt_3)Br$	Au(NHC)Cl
**Wavelength**	1.541	1.541
**Resolution range**	26.19 - 2.41 (2.496 - 2.410)	38.4 - 2.42 (2.506 - 2.42)
**Space group**	C 1 2 1	C 1 2 1
**Unit cell**	114.34 53.65 44.84<br>90 102.1 90	111.92 53.27 44.55<br>90 102.85 90
**Unique reflections**	9801 (944)	9385 (741)
**Multiplicity**	3.3 (3.1)	3.0 (1.2)
**Completeness (%)**	94.18 (91.83)	94.52 (74.65)
**Mean I/sigma(I)**	5.95 (1.05)	5.32 (1.01)
**Wilson B-factor**	52.65	43.68
**R-merge**	0.1862 (0.765)	0.1671 (0.5823)
**CC1/2**	0.981 (0.433)	0.976 (0.657)
**R-work**	0.2471 (0.3621)	0.2538 (0.3575)
**R-free**	0.2751 (0.3519)	0.2726 (0.2996)
**Number of non-hydrogen atoms**	2346	2348
**macromolecules**	2329	2329
**ligands**	2	1
**solvent**	15	18
**Protein residues**	301	301
**RMSD (bonds)**	0.002	0.003
**RMSD (angles)**	0.51	0.57
**Ramachandran outliers (%)**	0	0
**Average B-factors**	56.03	44.78
**macromolecule**	56.07	44.83
**ligands**	58.96	45.09
**solvent**	47.57	37.87
